# Conversion of β-carotene into astaxanthin: Two separate enzymes or a bifunctional hydroxylase-ketolase protein?

**DOI:** 10.1186/1475-2859-7-3

**Published:** 2008-02-20

**Authors:** Juan F Martín, Eduardo Gudiña, José L Barredo

**Affiliations:** 1 Institute of Biotechnology of León (INBIOTEC), Science Park, Av. Real 1, 24006, León, Spain; 2 University of León, Campus de Vegazana s/n, 24071, León, Spain; 3 R&D Biology, Antibióticos S.A., Avenida de Antibióticos, 59-61, 24009, León, Spain; 4 Lab. de Ciência e Tecnologia Alimentar. Departamento de Engenharia Biológica, Universidade do Minho, Campus de Gualtar, 4710-057, Braga, Portugal

## Abstract

Astaxanthin is a xanthophyll of great interest in animal nutrition and human health. The market prospect in the nutraceutics industries for this health-protective molecule is very promising. Astaxanthin is synthesized by several bacteria, algae and plants from β-carotene by the sequential action of two enzymes: a β-carotene, 3,3'-hydroxylase that introduces an hydroxyl group at the 3 (and 3') positions of each of the two β-ionone rings of β-carotene, and a β-carotene ketolase that introduces keto groups at carbons 4 and 4' of the β-ionone rings. Astaxanthin is also produced by the yeast-like basidiomycete *Xanthophyllomyces dendrorhous*. A gene *crtS *involved in the conversion of β-carotene to astaxanthin has been cloned simultaneously by two research groups. Complementation studies of *X. dendrorhous *mutants and expression analysis in *Mucor circinelloides *reveals that the CrtS enzyme is a β-carotene hydroxylase of the P-450 monooxygenase family that converts β-carotene to the hydroxylated derivatives β-cryptoxanthin and zeaxanthin, but it does not form astaxanthin or the ketolated intermediates in this fungus. A bifunctional β-carotene hydroxylase-ketolase activity has been proposed for the CrtS protein. The evidence for and against this hypothesis is analyzed in detail in this review.

## Review

Carotenoids are an important group of natural pigments with specific applications as colorants, feed supplements and nutraceuticals; they are also used for medical, cosmetic and biotechnological purposes. A few of the variety of natural and synthetic carotenoids available have been exploited commercially, including β-carotene, lycopene, astaxanthin, canthaxanthin, lutein and capxanthin [1-4]. Although more than 600 different carotenoids have been described from carotenogenic microorganisms [5], only β-carotene, lycopene and astaxanthin are commercially produced by microbial fermentation. These three compounds have various biological functions such as species-specific coloration, light-harvesting, photo-protection, antioxidant, and hormone precursor [6,7]. Dietary carotenoids have beneficial effects delaying the onset of many diseases such as arteriosclerosis, cataracts, age-related macular degeneration, multiple sclerosis, cardiovascular diseases, and some kinds of cancer [4]. For these reasons the demand and market of carotenoids have grown drastically [8].

## Production and use of astaxanthin

**Figure 1:**
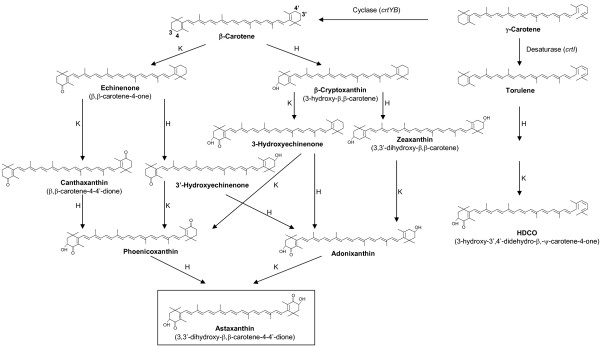
**Biosynthetic pathways for the conversion of β-carotene in different organisms.** K, β-carotene ketolase. H, β-carotene hydroxylase. From Álvarez et al., 2006, with permission.

Astaxanthin (Fig. 1) is a xanthophyll widely used as a pigment in aquaculture. The all-trans isomer is found in nature together with small amounts of 9-cis and 13-cis isomers [9]. Due to its high antioxidant activity, astaxanthin has also health benefits such as cardiovascular disease prevention, immune system boosting, bioactivity against *Helicobacter pylori*, and cataract prevention. Research on the health benefits of astaxanthin is recent and has mostly been performed *in vitro *or at the pre-clinical level with humans [10]. Research reports support the conclusion that a daily dose of ~5 mg of astaxanthin is of tremendous importance for health management, by protecting cells and body tissues from the oxidative stress caused by free radicals (singlet oxygen), among other reactive oxidants [11]. Astaxanthin producer companies have conducted several studies to demonstrate the safety of natural astaxanthin derived from *H. pluvialis*. A randomized, double-blind, placebo-controlled, 8-week trial designed to determine the safety of astaxanthin in 35 healthy adults revealed that healthy adults can safely consume 6 mg of astaxanthin per day [12]. Biotechnological production of astaxanthin using the yeast *Xanthophyllomyces dendrorhous *(the sexual state of *Phaffia rhodozyma*; [13]) or the alga *Haematococcus pluvialis *represent an advantage over the chemical synthesis or its extraction from crustaceans. Strain improvement programs to obtain astaxanthin overproducing *X. dendrorhous *strains [14] by gene cloning and manipulation [15-17] have been described. A new astaxanthin producing bacterium classified as *Paracoccus carotinifaciens *was described by Tsubokura et al. in 1999 [18]. This prokaryote presents a high speed of growth and an easier extraction process for the astaxanthin produced.

Nowadays natural β-carotene accounts for 15 to 20% of world demand [19]. A similar demand for natural astaxanthin is now emerging in the multi-billion dollar nutraceutical market.

## The xanthophylls biosynthetic pathway in *X. dendrorhous*

The biosynthetic pathway for astaxanthin has been studied in *X. dendrorhous *[20-23]. The conversion of common isoprenoid precursors into β-carotene is catalyzed by four enzymatic activities: (i) geranylgeranyl pyrophosphate (GGPP) synthase (encoded by the *crtE *gene), which converts farnesyl pyrophosphate and isopentenyl pyrophosphate into GGPP; (ii) phytoene synthase (encoded by the *crtYB *gene), which links two molecules of GGPP to form phytoene; (iii) phytoene desaturase (encoded by the *crtI *gene), which introduces four double bonds in the phytoene molecule to yield lycopene; and (iv) lycopene cyclase (encoded also by the *crtYB *gene), which sequentially converts the ψ acyclic ends of lycopene to β rings to form γ-carotene and β-carotene. The astaxanthin biosynthetic genes *crtE *[23,24], *crtI *[25], and *crtYB *[26] involved in the steps to β-carotene have been cloned from *X. dendrorhous *although no linear relationship between their transcript levels and carotenoid biosynthesis was found [24].

In bacteria, two additional enzymatic activities convert β-carotene into astaxanthin through several biosynthetic intermediates (Fig. 1): a ketolase which incorporates two keto groups at C-4 and C-4' in the molecule of β-carotene, and a hydroxylase which introduces two hydroxyl groups at C-3 and C-3'. The existence of a monocyclic pathway diverging from the bicyclic pathway at neurosporene and proceeding through β-zeacarotene, 3,4-didehydrolycopene, torulene and 3-hydroxy-3',4'-didehydro-β,ψ-carotene-4-one (HDCO) to the end product 3,3'-dihydroxy-β,ψ-carotene-4,4'-dione (DCD) has also been proposed [27].

The conversion of β-carotene into astaxanthin and other xanthophylls appears to proceed by different routes in *X. dendrorhous *as compared to photosynthetic bacteria, although the pathway in *X. dendrorhous *has not been completely elucidated yet (see below).

## β-carotene hydroxylases of the non-heme di-iron type

In general, hydroxylases introduce in their substrates an oxygen atom from molecular oxygen and require an electron donor to reduce the second oxygen atom to water. Two major groups of β-carotene hydroxylases are known: i) non-heme di-iron (NH-di-iron) hydroxylases that are related to fatty acid desaturases, and ii) cytochrome P-450 monooxygenases.

NH-di-iron hydroxylases have been described in bacteria, green algae and plants [28]. Phylogenetic analysis of this class of carotene hydroxylases revealed three distinct groups corresponding to a) plants and green algae, b) non-photosynthetic bacteria, and c) cyanobacteria. Although those three groups have little overall similarity, they all share four so-called histidine motifs (HX_4_H or HX_2_HH). These motifs are also characteristic of membrane-associated fatty acid desaturases and sterol desaturases.

The β-carotene hydroxylases of the NH-di-iron type require molecular oxygen, iron, ferredoxin and ferredoxin oxidoreductase for their activity; it seems that the histidine motifs are required for iron binding and are essential for the enzyme activity [29]. The histidine motifs are located in the hydrophylic domains of β-carotene hydroxylases and fatty acid desaturases [29]. In addition, there is a long conserved amino acid stretch HDGLVHXRXP (called motif 1), that overlaps with one of the H motifs, in all the NH-di-iron β-carotene hydroxylases. Interestingly, the β-carotene hydroxylase of the thermoacidophilic arquea *Sulfolobus solfataricus *maintains the consensus H motifs although the rest of the protein has little similarity to those of bacteria, plant and cyanobacteria.

All the β-carotene hydroxylases of this class show putative transmembrane helices suggesting that they are integral parts of the membrane systems [30,31]. However, the β-carotene hydroxylase of the alga *Haematococcus pluvialis *is not found in the chloroplasts and the conversion of β-carotene into astaxanthin takes place in cytoplasmic lipid vesicles, but not in the chloroplasts [32]. Expression studies of the β-carotene hydroxylase of *H. pluvialis *in *E. coli *indicate that it may use as substrates either β-carotene or ketocarotenoids such as canthaxanthin, to form respectively zeaxanthin or astaxanthin [32] (Fig. 1).

Some bacteria have β-carotene hydroxylases of the NH di-iron class, but with an enlarged range of substrates. The enzyme of *Paracoccus *sp. N81106 (previously classified as *Agrobacterium aurantiacum*), a marine bacterium that produces astaxanthin and adonixanthin [33,34], may use as substrates unsubstituted β-ionone rings and also their 4-keto derivatives [35]. The same is true for the β-carotene hydroxylases of *Pantoea ananatis *and *Pantoea agglomeran*s (previously classified as *Erwinia uredovora *and *Erwinia herbicola*, respectively) [36].

The myxol-producer marine bacterium *Flavobacterium *sp. [36,37] contains a β-carotene hydroxylase that shows low similarity to other bacterial NH di-iron β-carotene hydroxylases [38,39]. The enzyme of *Flavobacterium *sp. is a 3,3'-β-carotene hydroxylase which is able to convert β-carotene into zeaxanthin and astaxanthin.

In several plants (*Arabidopsis thaliana*, *Capsicum annuum*, *Citrus unshiu*) there are at least two β-carotene hydroxylases encoded by different genes [29,31,40,41]. The two hydroxylases of *A. thaliana *(encoded by *chy1 *and *chy2*) are similar and functionally redundant [42].

In summary, all the bacterial NH-di-iron hydroxylases have the ability to introduce hydroxyl groups in C-3 and C-3', but lack ketolase activity. Although all β-carotene hydroxylases of this class share a similar mechanism for introducing the oxygen atoms into β-carotene, they show differences in substrate specificity, thus widening the range of products with potential industrial interest.

## β-carotene hydroxylases of the cytochrome P-450 monooxygenase type

Several β-carotene hydroxylases belong to the cytochrome P-450 monooxygenase protein superfamily. This is a very large protein family including more than 5,000 monooxygenases distributed in all living beings. They include soluble and membrane-bound enzymes involved in a variety of catabolic processes and also in the biosynthesis of many secondary metabolites [43,44].

The enzymes of this class introduce in their substrates one of the oxygen atoms from molecular oxygen; the second oxygen atom is reduced to water with consumption of two reducing power equivalents [45]. The final donor of electrons for the P-450 monooxygenases is NAD(P)H. In most cases electrons are transferred from NAD(P)H to ferredoxin by a ferredoxin oxidoreductase that is encoded by a gene frequently linked to that of the P-450 monooxygenase [46]. In other systems the electrons are transferred from NAD(P)H to a diflavin reductase (containing FAD or FMN) and later to the P-450 monooxygenase. The molecular structure of several P-450 monooxygenases is known [47] and their catalytic mechanism is conserved despite low similarity between the different groups of enzymes in this family (see review by Werch-Reichhart and Feyereisen [45]).

Although the first cloned β-carotene hydroxylases were of the NH-di-iron type, in recent years several β-carotene hydroxylases of the P-450 monooxygenase class have been described in different bacteria [48]. We will limit the description in this review to that occurring in *X. dendrorhous*, in comparison with those of other organisms.

## Conversion of β-carotene into astaxanthin in different organisms

Two enzymatic activities convert β-carotene into astaxanthin through several alternative biosynthetic intermediates (Fig. 1): a ketolase which incorporates two keto groups at C-4 and C-4' in the molecule of β-carotene, and a hydroxylase which introduces two hydroxyl groups at C-3 and C-3'.

A β-carotene C-4 ketolase (encoded by *crtW*) has been found in marine bacteria (*Paracoccus *sp [35], *Brevundimonas *sp [38]), soil bacteria (*Bradyrhizobium *sp [49] and cyanobacteria) or encoded by the *bkt *gene in *Haematococcus pluvialis *[50,51]. These enzymes catalyze the direct conversion of a methylene group (in the 4 and 4' positions of the β-ionone ring of β-carotene) producing echinenone and canthaxanthin [52].

A recent mutational analysis of the β-carotene ketolase of *Paracoccus *sp. allowed to identify several features of this type of enzyme. This protein contains four transmembrane domains, similar to those found in fatty acid desaturases, and two other specific hydrophobic regions that might be involved in interactions with the carotenoid substrates [53]. In addition, it contains the aromatic amino acid WX_3_FX_3_Y domain occurring in many β-carotene ketolases and an aspartic acid rich sequence DDPDFD next to the second histidine motif [53].

The β-carotene ketolases encoded by the *bkt1 *and *bkt2 *genes of *H. pluvialis *convert β-carotene to echinenone and canthaxanthin, but does not accept zeaxanthin as a substrate [50,51], in contrast to the CrtW ketolase of *Paracoccus *sp.

A different group of β-carotene ketolases are represented by CrtO of *Synechocystis *sp. that introduce an asymmetric keto group in the molecule of β-carotene resulting in the formation of the monoketolated derivative echinenone [54]. These classes of ketolases are related structurally to the CrtI phytoene hydrogenases [55].

## The *crtS *gene of *X. dendrorhous *encodes a novel cytochrome P-450 hydroxylase involved in the conversion of β-carotene into astaxanthin

The biosynthetic pathway for β-carotene has been studied in *X. dendrorhous *[20-23]. As indicated above the conversion of common isoprenoid precursors into β-carotene in *X. dendrorhous *is catalyzed by four enzymatic activities. The GGPP synthase is encoded by the *crtE *gene. Two other genes encode the three remaining enzyme activities [56]. The *crtYB *gene encodes a hybrid protein with phytoene synthase-lycopene cyclase activities [25]. The *crtI *gene codes for a phytoene desaturase that catalyzes the introduction of four double bonds into phytoene yielding lycopene [26].

Recently, a gene (*crtS*) that encodes a novel cytochrome P-450 monooxigenase has been cloned simultaneously by two independent research groups [15,16]. This gene encodes a cytochrome P-450 monooxygenase that is involved in the conversion of β-carotene into astaxanthin and other xanthophylls.

**Figure 2:**
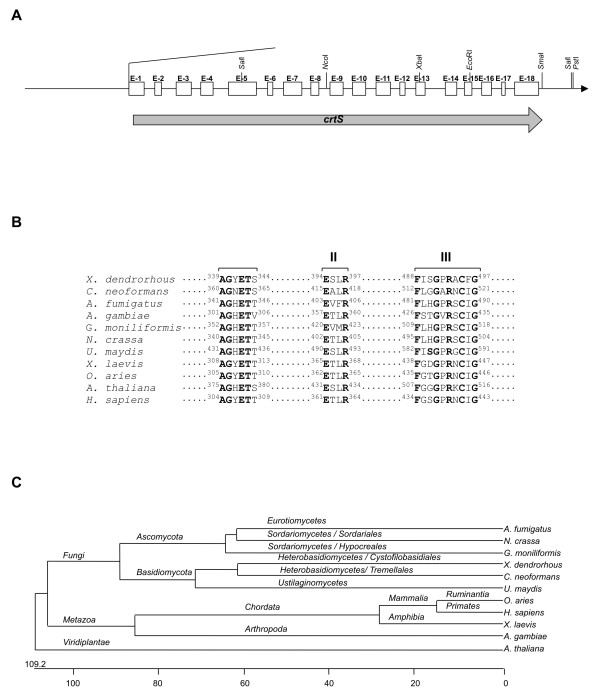
**Structure of the *crtS *gene of *X. dendrorhous *and characteristics of the encoded enzyme.** A) Organization of introns and exons of the *crtS *gene. The gene is shown by a thick arrow and the 18 exons are indicated by open boxes. Vertical arrows. B) Conserved motifs of CrtS and the P450 hydroxylases and C) Phylogenetic analysis of CrtS. Note the relationship with the homologous gene of heterobasidiomycetes. The units at the bottom of the figure indicate the number of substitution events (modified from Álvarez et al., 2006; with permission).

The *crtS *gene (named *asy *by Ojima and coworkers [16]) was cloned from the genomic libraries of two different strains *X. dendrorhous *VKPM Y2410 and ATCC 24203. In both strains there was a 3166 bp ORF consisting of 18 exons and 17 intron sequences (Fig. 2A). The *crtS *nucleotide sequence from *X. dendrorhous *VKPM Y2410 showed several changes when compared to the sequences from strains ATCC 2403 and ATCC 96594 (AX 034666). The changes in these strains do not affect the amino acid sequence of the CrtS protein [15].

**Figure 3:**
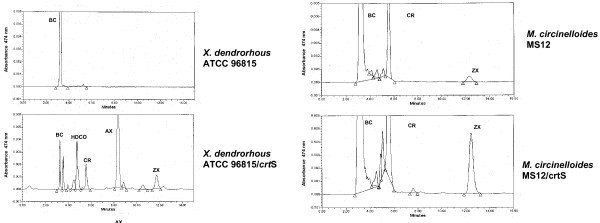
**HPLC analysis of β-carotene and xanthophylls produced by A) *X. dendrorhous *ATCC 96815 or B) *Mucor circinelloides *MS12, before and after transformation with the *crtS *gene.** CR, cryptoxanthin; ZX, zeaxanthin; AX, astaxanthin; HDCO, 3-hydroxy-3',4'-didehydro-β-ψ-carotene-4-one. Note that expression of the *crtS *gene in *M. circinelloides *results in the production of β-carotene hydroxylated derivatives only, whereas transformants of *X. dendrorhous *with the same gene produce astaxanthin in addition to the hydroxylated derivatives.

A different strain ATCC 96815 (strain Yan-1; [57]) that is known to be blocked in astaxanthin biosynthesis was found to contain two mutations: a G^1470^A change in the 5'-splicing region of intron 8 and a G^2268 ^insertion in exon 13 that causes a change in the reading frame [15,16]. This mutant strain accumulates β-carotene as shown by HPLC analyses (Fig. 3) and it is unable to synthesize cryptoxanthin or astaxanthin.

The encoded CrtS protein has a molecular weight of 62.6 kDa and shows strong similarity to other P-450 monooxygenases. The encoded protein contains the oxygen binding site (^339^AGYETS^344^) including the invariant G^340 ^and T^343 ^residues common to all cytochrome P450 proteins [58], the heme-binding domain (^488^FISGPRACFG^497^) thought to be involved in the folding of the heme-binding pocket [47], and a domain (^394^ESLR^397^) found in all cytochrome P450 proteins involved in the maintenance of the three-dimensional structure [59] (Fig. 2B). Conservation at the Cys^495^, which provides the thiolate ligand to the heme group [60], was also observed.

Phylogenetic studies revealed that the CrtS protein is closely related to cytochrome-P450 hydroxylases of fungi, especially to those from the Basidiomycota *Cryptococcus neoformans *and *Ustilago maydis*, and the Ascomycota *Aspergillus fumigatus*, *Giberella moniliformis *and *Neurospora crassa *(Fig. 2C). However, it showed insignificant identity to the *crtZ *gene product (β-carotene hydroxylase) involved in carotenoid biosynthesis in bacteria.

## Complementation of *X. dendrorhous *ATCC96815 and other mutants blocked in astaxanthin biosynthesis

Complementation of the strain ATCC 96815 with the *crtS *gene restored high levels of astaxanthin production, but also led to the accumulation of the intermediates cryptoxanthin (monohydroxylated derivative of β-carotene at C-3) and zeaxanthin (a 3,3'-dihydroxy-derivative of β-carotene). The production of a small amount of zeaxanthin, which is not formed in ATCC 96815, suggests that the CrtS enzyme performs hydroxylation in both β-ione rings (at the 3 and 3' positions). The lack of formation of the ketoderivative canthaxanthin (Fig. 3) suggests, in principle, that CrtS has no β-carotene ketolase activity; and that this activity may be provided by another enzyme(s) existing in *X. dendrorhous*. In addition ATCC 96815 transformed with the *crtS *gene produced HDCO (3'-hydroxy-3',4'-didehydro-β,ψ-carotene-4-one) that is not formed in the untransformed ATCC 96815 strain. The introduction of the *crtS *gene appears to trigger the action of other enzymes on the hydroxylated intermediates formed by the action of CrtS.

Complementation of the *X. dendrorhous *ATCC 96815 with the *crtS *gene has also been reported by Ojima et al. [16]. These authors observed that the formation of zeaxanthin is suppressed by addition of CrtS inhibitors, confirming the involvement of this enzyme in the hydroxylation of β-carotene.

It is clear from the experimental results that the CrtS P-450 monooxygenase is a β-carotene 3,3'-hydroxylase that introduces hydroxyl groups in either monocyclic or bicyclic intermediates resulting in the conversion of β-carotene to zeaxanthin (3,3'-dihydroxy-β-carotene). A ketolase activity could then convert zeaxanthin to astaxanthin. Ojima and co-workers [16] claimed that the CrtS P-450 monooxygenase may perform both the hydroxylase and ketolase reactions and, thus, it is able to convert directly β-carotene to astaxanthin. However, the evidence for this hypothesis is difficult to be interpreted and the final conclusion is dependent upon the presence or not of a separate ketolase activity in *X. dendrorhous *that works in cooperation with CrtS.

## Expression of *crtS *in *Mucor *reveals β-carotene hydroxylase activity, but not ketolase activity

To elucidate if CrtS catalyzes the synthesis of ketolated derivatives in addition to the hydroxylated β-carotene derivatives, the *crtS *cDNA was expressed in *Mucor circinelloides *under the control of the *carRA *promoter from *Blakeslea trispora*. Several transformants were shown to contain the intact expression construction. The untransformed *M. circinelloides *produces β-carotene as the main carotenoid and small amounts of the hydroxylated derivatives β-cryptoxanthin and zeaxanthin. The transformants with the *crtS *gene showed increased levels of β-cryptoxanthin and zeaxanthin (190 to 330%), but keto-derivatives such as canthaxanthin, echinenone or astaxanthin were not detected [15]. This result supports the presence of β-carotene hydroxylase activity in CrtS, but argues against the existence of a ketolase activity in this protein. *M. circinelloides *appears to lack a separate functional β-carotene ketolase activity and this activity is not provided in the heterologous system by CrtS.

The bacterial *crtZ *and *crtW *genes were used to transform *M. circinelloides *[61], showing that *M. circinelloides *when transformed with the CrtW ketolase is able to form echinenone, canthaxanthin and a small amount of astaxanthin, thus confirming the ability of this fungus to synthesize β-carotene keto-derivatives when a ketolase activity is provided.

## The β-carotene hydroxylase-ketolase bifunctional protein hypothesis

This hypothesis is based in the introduction in *X. dendrorhous *PR-1-104 (a β-carotene accumulating mutant having an uncharacterised mutation(s); [62]) of the separate bacterial *crtZ *(encoding a β-carotene hydroxylase) or the *bkt *gene from *H. pluvialis *(encoding a β-carotene ketolase; [52]). HPLC analyses of the transformants revealed that *X. dendrorhous *PR-1-104 [*crtZ*] transformants produced the hydroxylated β-carotene derivatives cryptoxanthin and zeaxanthin, whereas clones transformed with the *bkt *gene produce the keto-derivatives 4-ketotorulene, echinenone and canthaxanthin. Complementation of the same mutant PR-1-104 with the *crtS *gene results in a mixture of hydroxylated and ketolated derivatives. According to these results, Ojima and co-workers [16] claim the presence of both enzymatic activities in the CrtS protein. However, it is also possible that each bacterial gene encoding either a β-carotene hydroxylase or a ketolase are expressed forming enzymes that can not interact with an endogenous *X. dendrorhous *complementing enzyme.

Moreover, the non-hydroxylated derivatives, such as echinenone and canthaxanthin, are accumulated in *X. dendrorhous *when the CrtS activity is inhibited with cytochrome P450 hydroxylase inhibitors [16]. This result is difficult to explain if there is a single enzyme involved in the conversion of β-carotene to astaxanthin and suggests that two enzymes, with P450-hydroxylase activity and ketolase activity respectively, are involved in the conversion of β-carotene into astaxanthin in *X. dendrorhous*. Whether both activities belong to CrtS is an open question, since a separate ketolase (so far uncloned) may occur in *X. dendrorhous*. The definitive answer to this question will only be known when the CrtS enzyme is purified and *in vitro *studies are performed. Attempts to gain information by expressing a combination of three genes in *E. coli *were not conclusive due to the complexity of the three plasmid system used and the poor expression of the *crtS *gene in this bacterium [16].

**Figure 4:**
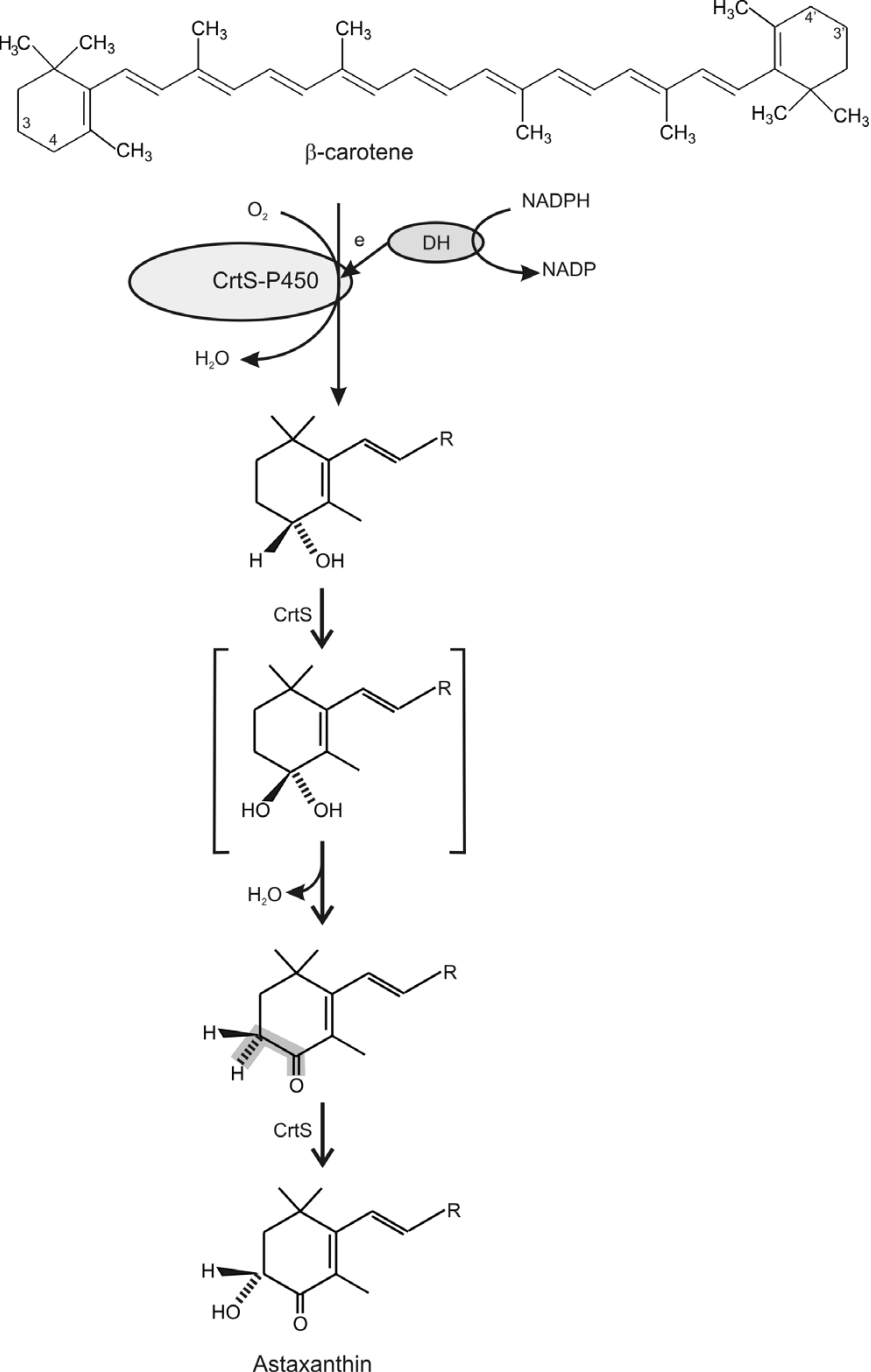
**Biosynthetic steps in the conversion of β-carotene into astaxanthin by a bifunctional CrtS protein as proposed by Ojima et al.** (2006). Only half of the β-carotene molecule is shown for simplicity.

A reiterated action of the CrtS hydroxylase activity on β-carotene including a dehydration activity has been proposed by Ojima and co-workers [16] (Fig. 4). In this model, the conversion of β-carotene into astaxanthin starts with an hydroxylation at C-4 of one of the ionone rings of β-carotene that is later dihydroxylated and, by removal of a water molecule, is converted to the 4-keto derivative (echinenone) that is subsequently hydroxylated again by CrtS at C-3 (3-hydroxyechinenone) and finally converted in phoenicoxanthin and astaxanthin. It is unclear if all those steps are carried out by a single CrtS enzyme in *X. dendrorhous*. Other β-carotene hydroxylases (or ketolases) different from CrtS occur and have been cloned recently from *X. dendrorhous *(E. Gudiña and J. F. Martín, unpublished results).

## Future prospects

The growing worldwide market value of carotenoids is projected to reach over US 1 billion by the end of this decade [63]. The nutraceutical companies have integrated carotenoids in their development strategies based on the claim of their proven antioxidant properties. Synthetic astaxanthin dominates the world market but recent interest in natural sources of the pigment has increased substantially. According to Pfander [64], the price in the market for a dispersion containing 5 to 10% of carotenoid is about 600 $/kg for β-carotene, 1300 $/kg for canthaxanthin, and 2500 $/kg for astaxanthin. Nowadays, most of the commercial astaxanthin for aquaculture is produced synthetically from petrochemical sources, with an annual turnover of over $200 million, and a selling price of about 2000 $/kg of pure astaxanthin. The interest for the microbiological production of carotenoids has increased during the last years due to increasing demand for the aquaculture market, especially for nutritional salmonids and shrimps.

Understanding the conversion of β-carotene into astaxanthin will open the door for a rational amplification of the last steps of xanthophylls production, either in *X. dendrorhous *or in alternative microbial systems like the fungus *Blakeslea trispora *that is used at present for large scale β-carotene production but lacks the genetic information for conversion of β-carotene into xanthophylls [65]. The possibility of producing β-carotenoid compounds in the yeast *Saccharomyces cerevisiae *using the genes from *X. dendrorhous *has also been recently explored [66].

## Conclusion

In conclusion, the conversion of β-carotene into astaxanthin in different organisms takes place by the combined action of a β-carotene hydroxylase and a β-ketolase that work successively on different intermediates, resulting in a metabolic grid of reactions and intermediates (Fig. 1). This wealth of intermediates is of biotechnological interest because it provides new xanthophylls for the animal nutrition and human health industries. In *X. dendrorhous *both activities have been reported to be encoded in a single bifunctional enzyme [16], but this hypothesis has not been confirmed by other laboratories [15]. Further biochemical evidence is required to elucidate if a single protein contains the two activities or whether there are separate β-carotene hydroxylase and ketolase activities encoded by two separate genes, only one of which (*crtS*) has been cloned, in this yeast-like basidiomycete.

## Abbreviations

GGPP: geranylgeranyl pyrophosphate; HDCO: 3-hydroxy-3',4'-didehydro-β,ψ-carotene-4-one; DCD: 3,3'-dihydroxy-β,ψ-carotene-4,4'-dione; NH: non-heme.

## Competing interests

The author(s) declare that they have no competing interests.

## Authors' contributions

JFM has organized this article and contributed most of the written text. EG performed the experimental work on the characterization of the *X. dendrorhous crtS *gene in one of the referenced articles. JLB contributed parts of the article dealing with the industrial and medical relevance of β-carotene and astaxanthin. All authors read and approved the final manuscript.
